# Evaluating implementation and impact of a provincial quality improvement collaborative for the management of chronic diseases in primary care: the COMPAS+ study protocol

**DOI:** 10.1186/s12875-019-1072-y

**Published:** 2020-01-07

**Authors:** Brigitte Vachon, Isabelle Gaboury, Matthew Menear, Marie-Pascale Pomey, Denis Roy, Lise Houle, Mylaine Breton, Arnaud Duhoux, Valérie Émond, Guylaine Giasson, Janusz Kaczorowski, France Légaré, Marie-Thérèse Lussier, Pierre Pluye, Alain Vanasse

**Affiliations:** 1 School of Rehabilitation, Faculty of Medicine, Université de Montréal and Centre de recherche du CIUSSS de l’Est-de-l’Île-de-Montréal, Montreal, Quebec, Canada; 2 Faculty of Medicine and Health Sciences, Université de Sherbrooke, Sherbrooke, Canada; 3 Family Medicine and Emergency Medicine, Université Laval, Quebec, Canada; 4 Public Health School, Unversité de Montréal, Montreal, Canada; 5 Institut national d’excellence en santé et en services sociaux, Montreal, Canada; 6 Faculty of Nursing, Université de Montréal, Montreal, Canada; 7 Institut national de santé publique du Québec, Quebec, Canada; 8 Faculty of Medicine, Université de Montréal, Montreal, Canada; 9 Faculty of Medicine, McGill University, Montreal, Canada

**Keywords:** Quality improvement, Primary care, Chronic disease management, Knowledge translation, Diabetes, Chronic pulmonary obstructive disease

## Abstract

**Background:**

Chronic conditions such as diabetes and chronic obstructive pulmonary disease (COPD) are common and burdensome diseases primarily managed in primary care. Yet, evidence points to suboptimal quality of care for these conditions in primary care settings. Quality improvement collaboratives (QICs) are organized, multifaceted interventions that can be effective in improving chronic disease care processes and outcomes. In Quebec, Canada, the Institut national d’excellence en santé et en services sociaux (INESSS) has developed a large-scale QIC province-wide program called COMPAS+ that aims to improve the prevention and management of chronic diseases in primary care. This paper describes the protocol for our study, which aims to evaluate implementation and impact of COMPAS+ QICs on the prevention and management of targeted chronic diseases like diabetes and COPD.

**Methods:**

This is a mixed-methods, integrated knowledge translation study. The quantitative component involves a controlled interrupted time series involving nine large integrated health centres in the province. Study sites will receive one of two interventions: the multifaceted COMPAS+ intervention (experimental condition) or a feedback only intervention (control condition). For the qualitative component, a multiple case study approach will be used to achieve an in-depth understanding of individual, team, organizational and contextual factors influencing implementation and effectiveness of the COMPAS+ QICs.

**Discussion:**

COMPAS+ is a QI program that is unique in Canada due to its integration within the governance of the Quebec healthcare system and its capacity to reach many primary care providers and people living with chronic diseases across the province. We anticipate that this study will address several important gaps in knowledge related to large-scale QIC projects and generate strong and useful evidence (e.g., on leadership, organizational capacity, patient involvement, and implementation) having the potential to influence the design and optimisation of future QICs in Canada and internationally.

## Background

Preventing and managing chronic diseases represents a massive challenge to healthcare systems. Globally, chronic diseases figure among the most important causes of mortality and morbidity and impose a toll on individuals and populations that can last for years or decades [1]. In Canada, three in five adults over the age of 20 live with a chronic disease, while four in five adults have at least one modifiable risk factor for chronic illness [2, 3]. Two thirds of all deaths each year are caused by four major chronic diseases: cancer, cardiovascular diseases, diabetes, and chronic obstructive pulmonary disease (COPD) [3]. Such chronic diseases adversely impact the lives of millions of Canadians and represent a significant burden to the healthcare system, accounting for over one third of all direct healthcare expenditures and billions of dollars in indirect costs due to lost productivity [2–4].

There is a strong consensus in Canada and internationally that primary care has a critical role to play in the prevention and management of chronic diseases [4–9]. These services act as an important hub for comprehensive care that connects patients to other specialists and community services [10]. Recent reforms to strengthen preventive and team-based care in Canadian primary care services offer opportunities to improve outcomes for people with chronic diseases [4, 5, 11]. Yet, there is substantial evidence suggesting that the potential of primary care to effectively prevent and manage chronic conditions has not been realized and that many patients struggle to access high-quality chronic disease care [4, 5, 12–18].

In the past two decades, many strategies to improve the quality of healthcare have emerged and the evidence base for these strategies is growing rapidly [19]. Quality improvement (QI) strategies may include a range of activities and target individual patients or providers, teams, organizations, and/or broader health systems or networks [19, 20]. Previous systematic reviews have shown that QI projects may be more effective when they are multi-faceted and target multiple levels or stakeholders [21, 22]. Strategies targeting multiple levels of change have taken various forms, including quality assurance processes, service re-engineering, and disease management or clinical governance programs [19, 23–26].

One kind of multifaceted and multi-level intervention that is gaining in popularity is the quality improvement collaborative (QIC). A QIC involves multidisciplinary groups of clinicians and managers that participate in a structured process to identify best practices and change strategies, apply improvement methods, report results, and share information about ways of achieving improvement [27, 28]. Although models vary, QICs typically address a specific healthcare topic, involve multiple clinical teams from multiple sites, integrate a series of structured activities (e.g. meetings, workshops, audit and feedback, activities to promote collaboration), and encourage rapid cycle changes consistent with the Model of Improvement [19, 27]. In addition, a group of expert change agents often support participants by bringing together the scientific evidence, sharing practical knowledge and advice on best practices and QI methods, and facilitating implementation of strategies to improve care [19, 27]. A recent systematic review of 64 studies found that QICs resulted in a significant improvement in at least one primary outcome measure in 83% of studies, and in 85% of studies conducted in primary care [29]. A small group of studies included in the review additionally reported that QICs could be cost effective or lead to cost savings, and that intervention effects could be sustained 6 to 24 months after the end of the QIC.

While QICs are becoming increasingly widespread, important knowledge gaps for this strategy persist. Five systematic reviews on various aspects of QICs (e.g. effectiveness, components, determinants of success) [27–31] have pointed to numerous areas where new knowledge for this strategy is urgently needed. Wells and collaborators [29] have also noted that few papers provide enough detail to fully understand QIC interventions and that approaches vary considerably from one study to another. Most studies report using the Model for Improvement but it remains very difficult to determine with how much fidelity and intensity the model was implemented. Furthermore, many studies were uncontrolled before-after studies and lacked pre-intervention data before starting the intervention.

Currently, a large-scale QIC initiative known as COMPAS+ is being undertaken in the province of Quebec, Canada to improve the quality of chronic disease care in primary care settings. The fruit of a formal partnership between the Quebec Ministry of Health and Social Services (MSSS) and the *Institut national d’excellence en santé et en services sociaux* (INESSS), the provincial advisor on clinical excellence in health and social care, COMPAS+ is the first integrated clinical governance program to be implemented in the province. INESSS has the mandate to roll out COMPAS+ across the province, starting with a focus on two chronic conditions: diabetes and COPD. It is thus essential that a robust evaluation accompany this initiative, both to better understand the implementation processes and to assess the outcomes of the QICs and conditions necessary for their eventual scale-up to new chronic diseases and care settings.

### Development and previous evaluation of the COMPAS+ program

The QICs being investigated in the current study are based on an initial demonstration project conducted in Quebec’s Montérégie region (2008–2014) called the COMPAS (*Collective for best practices and improvement in primary care services*) program [32]. The program’s main goals were to engage primary care providers in continuous QI, improve interprofessional collaboration, and enhance the quality of care for priority chronic diseases [32]. The innovation central to COMPAS was the way in which it combined the use of population-based data to provide feedback on chronic disease performance, engaged participants in a process of critical reflection around their performance, and facilitated the development of QI action plans through a process of collaborative problem solving [32, 33]. These components were delivered as part of a half-day continuing professional development workshop for primary care teams from a same territory. COMPAS integrated notions of the Chronic Care Model [34] and Model for Improvement [35] and was guided by a well-articulated program theory of change [32, 33]. An evaluation of 10 workshops on diabetes management [33] revealed that COMPAS was effective in engaging participants and helping them recognize gaps between current practice and those outlined in clinical guidelines. QI action plans very frequently targeted improvement of interprofessional collaboration and addressed areas such as primary and secondary prevention of diabetes, screening practices, supporting patient self-management, and enhancing patients’ access to evidence-based treatments. However, only 25% of teams’ QI plans were fully implemented due to barriers such as a lack of time, local leadership, and supports for QI from their organizations.

The promising results of the COMPAS program led to its adoption in 2015 as a priority project by the Quebec’s Ministry of Health and Social Services. INESSS partnered with the research team and four health regions to develop and pilot an enhanced version of the program, called COMPAS+. In 2016 and 2017, new COMPAS+ QICs for COPD and diabetes were launched that built on the initial program components but also included new elements. Among the improvements was the extension of reflective practice workshops to a full day, the obtaining of formal institutional approval for the program from leaders in each region, and the creation of local implementation teams that now received long-term post-workshop supports from QI experts to facilitate the achievement of local QI projects. Furthermore, INESSS established patient involvement in COMPAS+ program governance and began involving local patient partners in workshops. Observations from this pilot phase of COMPAS+ suggest that participants are successfully engaging in the program and producing action plans, and that these plans have received higher levels of institutional support than seen previously. Still, many implementation processes and outcomes of the QICs have yet to be examined in depth.

### Study objectives

The overall aim of this study is to evaluate implementation and impact of COMPAS+ QICs for chronic diseases prevention and management in primary care. The specific objectives are to:

1. Evaluate the extent to which COMPAS+ is more effective than feedback alone in improving the quality of care offered to people with chronic diseases, notably diabetes or COPD.
2. Evaluate the extent to which COMPAS+ supports the implementation of QI projects, the improvement of interprofessional collaboration and the integration of a culture of continuous QI in primary care.
3. Describe variations in the implementation of the COMPAS+ intervention and document the influence of contextual factors on the effects of the intervention.

## Methods

### Conceptual frameworks

The main conceptual framework guiding this study is Brennan’s Informing Quality Improvement Research (InQuIRe) in primary care framework [36, 37]. Based on a systematic review, this comprehensive framework outlines [1] contextual factors at organization, team and individual levels that influence QI success, [2] mediating factors in the QI process, such as organizational readiness for change, [3] key elements of the QI process (QI methods, teamwork), and [4] proximal and distal outcomes of QI. The study also benefits from the program theory that was developed as part of the initial COMPAS program [32]. This program theory identifies the program activities, moderating factors, and outcomes specifically thought to underpin the COMPAS collaboratives. Finally, the study is guided by the Montreal Model of patient engagement, which describes how patients can be engaged in the organization and improvement of healthcare, along a continuum of engagement [38]. We strive to involve patients as true partners throughout all phases of COMPAS+ and its evaluation, and the primary patient partner (LH) has informed the objectives and methods of this proposal.

### Study design

The implementation and evaluation of COMPAS+ is embedded in an integrated knowledge translation (KT) approach. This means that all relevant stakeholders are involved in the decisions and the conduct of the different research phases. INESSS has already put in place several mechanisms (steering and implementation committees) to ensure that decision makers, clinicians, researchers and patient representatives are meaningfully engaged in COMPAS+ governance and every step of QIC development, implementation and evaluation, thus ensuring relevance and usefulness of results [39].

To achieve the study objectives, a mixed method convergent (concomitant) study design [40] will be used where the quantitative component of the study is a controlled interrupted time series (ITS) [41] and the qualitative component is a multiple case study [42]. Firstly, to asses whether COMPAS+ is more effective than feedback alone in improving the quality of services offered to people with a chronic healthcare condition, we will conduct an ITS – the most common evaluation approach used for large-scale QI collaboratives projects [29]. It represents an ideal choice of design in our circumstances given the iterative nature of local QI projects and the integrated healthcare organizations’ need for flexibility in the roll out of the QICs. Secondly, a convergent multiple case study [42] will allow our team to achieve a deep understanding of the implementation of COMPAS+, of the effectiveness of the QICs, and the factors influencing the implementation and effectiveness of the COMPAS+ intervention in different contexts. Case studies are an ideal approach for the in-depth examination of the dynamic contexts and complex processes involved in implementing collaboratives in real-world primary care settings [43]. The cases are each of the large integrated healthcare organizations participating in the study.

### Quantitative component: controlled interrupted time series

#### Setting, participants, randomization

Working in collaboration with INESSS, we will invite integrated health and social service centres to participate in the study. These integrated centres are responsible for ensuring accessibility, continuity, and quality across a continuum of health services (primary and specialist care) for the population in their region [44]. Integrated centres include all public healthcare organizations, such as hospitals, local community health and social services centres, long-term care facilities, and rehabilitation centres under a single governing body per territory. There are 22 integrated centres in the province of Quebec. Given that five had already piloted the COMPAS+ program, 17 integrated centres are eligible for this study. Each centre establishes service agreements with medical clinics in their region. Family Medicine Groups (FMGs) are the main model for interdisciplinary team-based primary care, with over 330 FMGs providing care to nearly 80% of the population. These FMGs work in collaboration with other health services provided in the community based on their geographical proximity.

We will identify nine integrated centres and stratify them according to the type of region and the size of the population served (e.g. rural (less than 150,000 inhabitants in the main city), semi-rural (150 to 200,000 inhabitants in the main city) or urban (more than 200,000 inhabitants in the main city)). They will be randomly assigned within strata to one of the two study groups using random assignment generator: 1) *control group* consisting of three centres receiving only a feedback intervention and 2) *experimental group* consisting of six centres receiving the COMPAS+ intervention in addition to the feedback intervention [41].

#### Feedback alone intervention

The feedback intervention will take the form of a summary sheet that presents the results of a set of performance indicators extracted from administrative databases currently available to INESSS. The choice of the indicators for the summary sheet emerged from a consensus between policy makers, managers, healthcare professionals and patients. Quality indicators were produced using data from the Institut national de santé publique du Québec and INESSS, the latter having access to 11 linked health administrative databases. These indicators present a portrait of a specific clientele care (e.g. diabetes or COPD) at the local service network level, the integrated centre level, and the provincial level to allow comparison. A local service network is an integration of health and social services in a given territory responsible to provide a set of services for the population of that specific territory. Integrated centres are usually composed of a few local service networks. The feedback summary sheet will be developed in collaboration between INESSS and the research team. It can be used by the integrated centres to analyze current practices and identify local or regional QI needs. It will be sent to all directors of participating institutions as well as to relevant middle managers and chiefs of all family medicine groups in the region. For the control group, no further QI support will be provided.

#### COMPAS+ quality improvement collaboratives

For each integrated centre receiving the COMPAS+ intervention, reflective practice workshops will be offered in three local service networks. For each local service network, it is expected that approximately 30 people will participate in the COMPAS+ workshop (managers, health professionals, patient partners) and that, after the workshop, a local QI project implementation committee will be set up to implement action plan(s).

The COMPAS+ QICs will be conducted in three phases: 1) a preparatory phase, 2) a workshop phase, and 3) QI project follow-up phase.

##### 1) Preparatory phase

During this phase, INESSS will obtain formal support for the COMPAS+ program from integrated healthcare organization directors and help from local implementation committees within this organization to encourage strong and sustained local leadership before and after QI reflective workshops. Local implementation committees (managers, health professionals and patient partners) will facilitate the organization of workshops, participant recruitment, and QI project implementation and monitoring. Primary care teams – consisting of family physicians, nurses, other professionals, and managers of the same local service network – will be invited to participate in the QI reflective workshop, as will three or more patient partners living with the targeted chronic disease.

##### 2) QI reflective workshop

The workshop will feature three core QI components: 1) A tailored feedback intervention that includes a population-based performance assessment and comparison to best practices outlined in clinical practice guidelines; 2) Critical reflection exercises in large and small groups to promote reflection around the selected quality indicators; 3) A collaborative problem solving and action planning process involving small groups of 8–10 people that identify priority problems, propose actionable solutions, and negotiate the content of their QI action plans. External experts in QI and change management working with INESSS will facilitate the workshops, share information about best practices in QI and chronic disease care, and guide participants through the workshop’s QI activities. Patient partners will actively participate to the workshop and help shape the teams’ QI action plans. In order to properly support the patient partners during the process, a patient coach partner will meet with patient partners during their preparatory training prior to the workshop and provide guidance, encouragement, and role modelling during the workshop.

##### 3) QI project follow-up phase

After the workshop, the local implementation committee will meet with an INESSS QI team a minimum of four times over the next 18 months to finalize or refine local QI action plans and review progress with the implementation of QI projects. These meetings will actively engage local committees in the appropriate use of the Model for Improvement and use (a) active facilitation strategies to clarify QI objectives and activities and facilitate repeated small-scale improvement cycles, and (b) process control charts - a visual tool that displays objectives, indicators, activities and responsibilities - are used to support the team [45].

#### Outcomes measures

To assess the effectiveness of the COMPAS+ QICs, we will use three main measures of primary care quality easily retrievable from INESSS’s health administrative databases: [1] the number of emergency visits for ambulatory care sensitive conditions, a widely used measure of primary care accessibility and quality [46, 47], [2] number of patients visiting five or more different physicians over a period of twelve months, because it is an indicator of how formal the relationship between a family physician and a patient is (patient rostering has been recognized as an important feature of a high-performing primary care system) [48–50]; and [3] persistence to medication regimens over a period of twelve months, since poor adherence compromises effectiveness of treatment and health of patients at risk of chronic disease complications [51, 52].

#### Interrupted time series power calculation and analysis

The lack of estimates for some parameters required for the calculation of an ITS study sample precludes the calculation of a precise sample size. However, 36 points of observation (12 months before, 24 months after) should provide > 80% power to detect a significant effect of the intervention on primary outcome measures, assuming an effect size > 0.5 (medium effect or greater), autocorrelation > − 0.1, and a first-order autoregressive segmented regression model with both level (intercept) and trend (slope) changes expected [53]. To isolate the effect of the COMPAS+ QIC on the three quality indicators from other changes that could take place during the study period while adjusting for time, seasonal trends, and random errors, differences between the two study conditions (COMPAS+ or feedback only) will be assessed using linear mixed models for longitudinal data, where individuals’ data observed over time are nested into QICs to take account of the inter-QIC correlation. Descriptive statistics will also be generated for both study groups and all QICs.

### Qualitative component multiple case study

#### Setting and participants

The nine integrated health centres recruited to participate in the ITS study will also participate in the multiple case study. Baseline and longitudinal data will be collected to provide a rich description of each control and intervention site context and impacts of the received intervention. Between 7 to 10 key informants will be recruited for each site (senior managers and middle chronic disease and primary care managers) to participate in interviews. COMPAS+ workshops participants (managers, professionals, patients) and local implementation teams will also participate in the multiple case study.

### Data collection

#### Baseline assessment of control and intervention groups

Baseline interviews will be conducted for each case. The purpose of these interviews is to establish a portrait of the current organization of services and quality of care for people with the targeted chronic disease and to assess the extent to which QI and transformation projects have previously been implemented or are currently underway or planned in the region. This baseline evaluation will shed light on the adoption of best practices in chronic disease care by integrated health centres and their partners, help identify contextual factors at play in each region, and provide a basis for comparing services before and after the COMPAS+ QICs. Semi-structured individual and/or group interviews will be conducted with key informants (*n* = 7 to 10), including senior and middle managers responsible for chronic diseases and primary care services.

### Documenting implementation of QI projects

#### Control group

Following the feedback alone intervention, interviews will be conducted with the same key informants interviewed at baseline in each control site to document initiation of any QI projects or changes brought to services for the targeted chronic disease. Interviews will also capture the ways in which the feedback intervention supported or not the implementation of these projects or changes. Key informants will also be asked to share documents or reports that may be relevant to the QI projects.

#### Intervention group

To measure if COMPAS+ results in the initiation and implementation of QI projects, the local implementation committees will complete a project charter every four months. It will provide information on the content and level of implementation of QI projects. The tool developed by Lemire & Litvak [54] will be used, though Lean A3 reports [55] may be utilized as an alternative when already in use by the participants. During the follow-up phase of the QI project, local committee meetings will be recorded, and we will maintain a log (field notes) to continuously document the QI processes used by these committees. The level of achievement of the local QI projects will be assessed using Goal Attainment Scaling (GAS) [56] at 12 and 24 months after the intervention. GAS is an appropriate method to assess programs that have an individualized approach to intervention planning and was shown to be a reliable, valid and responsive method to document the impact of QI programs [57, 58]. GAS scales will be developed in collaboration with each local implementation committee. Any relevant documentation relevant to the QI projects will also be collected.

### Documenting secondary outcomes, levels of implementation and influencing factors for the intervention group

Data will be collected from individual health professionals and managers who participated in the COMPAS+ workshops to document the pre- and post-workshop levels of innovative culture (18 items) [59, 60], willingness and preparation for change (13 items) [60, 61], interprofessional collaboration (20 items) [62, 63], communication (5 items) [64], and readiness for patient engagement (5 items) [65]. These are validated scales that have been used in other studies evaluating QICs. They were pre-tested during the pilot phase of COMPAS+ and their scores revealed pre-intervention differences between sites. Data on these measures will be collected at the time of the workshops and at 12 and 24 months post-workshop through an online survey platform.

To document implementation levels and factors influencing implementation, field notes will be kept by the research team to document all meetings with the local implementation committees and interactions with local stakeholders, thus capturing vital information on QIC implementation and impacts throughout the course of the study. A structured grid will be used to track if the delivery of the three intervention phases went as planned and whether and how the support provided by the COMPAS+ team varied across sites.

Finally, group interviews, lasting one to three hours, with the local implementation teams of each large integrated healthcare organization will be conducted by a research assistant at 24 months of follow-up based on the Inquire Framework [36] to document the individual, team, organizational, and contextual factors that influence QI process.

#### Multiple case study data analysis

The framework method [66] will be used to analyse the qualitative and descriptive quantitative data collected for each integrated health centre and QIC. The advantage of this method is that it was developed for use in large-scale applied health research and is very useful to compare data across cases as well as within individual cases. It is composed of 7 stages: 1) transcription, 2) familiarisation with the interview, 3) coding, 4) developing a working analytical framework, 5) applying the analytical framework, 6) charting data into the framework matrix and 7) interpreting the data. In this approach, the coding aims to classify all the data so it can be compared systematically with other part of the data set. Inductive and deductive approaches (informed by the INQUIRE framework and COMPAS program theory) will be used. After coding a few cases, an analytical framework will be developed and apply to the other cases. Several iterations of the framework will be required. After applying the analytical framework to all the cases, a spreadsheet will be used to generate a matrix and the data will be charted into the matrix. Charting ensures paying close attention to each case before moving to interpretation and conduct cross-case analysis [66]. The analysis will ultimately allow us to produce narrative reports describing the story of each integrated health centre’s experience with the COMPAS+ or feedback interventions.

### Integration of the quantitative and qualitative study components

Based on the framework developed by Pluye and collaborators [67], a combination of two types of mixed methods analysis strategies will be used: comparison of QUAN and QUAL results obtained separately and assimilation of QUAN data into QUAL data. First, quantitative data collected for the ITS study will be added to the matrix and integrated in a single visual [40]. Multiple repeated quantitative baseline and follow-up data collected for the ITS will be compared to qualitative data collected during interviews to provide a thorough description of each study site. Convergence and divergence between qualitative and quantitative results will be assessed. Afterwards, quantitative data will be transformed into qualitative data to be analysed with the qualitative data. Based on the integration of both kinds of results, study sites will be categorized as high, moderate or low at implementing QI projects whether they are intervention or control sites. Characteristics and factors between cases will be identified and explanations for differences in the level of implementation, in the influence of contextual factors and possible interaction with achieved outcomes will be generated to provide clear answers to objectives 2 and 3 of this study. Two experienced qualitative researchers will conduct these analyses, in close collaboration with our patient partners and interdisciplinary research team that will meet regularly during the analyses phase.

## Discussion

COMPAS+ is a QI program that is unique in Canada due to its integration within the governance of a provincial healthcare system and its capacity to reach many primary care providers and people living with chronic diseases across the province. The evaluation that will accompany the implementation of QICs will address major knowledge gaps in the implementation and effectiveness of these large, complex programs. Indeed, strong evidence generated from the project on issues such as leadership, organizational capacity, patient involvement, and implementation supports could significantly influence the design and optimisation of future collaboratives in Canada and around the world. Such information will also be critical for shaping the strategic directions and broader implementation of the COMPAS+ program in Quebec.

Several challenges and limitations of the study should however be mentioned. Among the biggest challenges is in the identification and use measures to capture the real-world impacts of the QICs given that participants will develop QI action plans during and after the reflective practice workshops. These QI projects can take many forms and it is not possible in advance to know what improvement objectives or activities will be pursued. While we have already identified several QI process, organizational or service related outcomes to measure throughout the study, other relevant measures may only become apparent as the study progresses and may not be applicable in all settings. This is also a pragmatic study conducted in real-world healthcare settings and as such it is imperative that our research methods consider the time and resource constraints of our partners. The communication channels put in place with our integrated KT approach have already been helpful in this regard. Finally, at this stage, the majority of quantitative and qualitative data will be collected from health professionals and managers involved in the QICs. However, the ultimate beneficiaries of COMPAS+ are people living with chronic diseases and it would be ideal to incorporate a data collection from these partners in the future.

This study has also several strengths. First, we are using an integrated KT approach with a strong, meaningful and sustained engagement from project partners, including clinicians, decision-makers and patient partners. We are also using a mixed methods approach with rigorous quantitative (interrupted time series) and qualitative (multiple case study) components. Much of the previous research on QICs has involved uncontrolled before-after studies that are limited in their ability to mitigate for bias and confounding and consider contextual factors having a potential influence on outcomes targeted by the QIC (Wells et al. 2018). Even when designs like ITS were used, previous studies have lacked enough repeated observations to adequately describe trends in outcomes (Wells 2018). The present study compares COMPAS+ to a feedback only control condition, gathers data through multiple sources at multiple time points before the intervention and for 24 months post-intervention, and adopts a mixed approach to data analysis to ensure a rich understanding of implementation processes and factors contributing to intervention effectiveness. Finally, the random allocation of sites to either the intervention or control conditions is another strength of this research.

Since QICs are increasingly being used and implemented to support QI efforts in healthcare, we expect that the results of this study will produce significant and important knowledge to inform the development and evaluation of future QICs and implementation of innovative chronic disease management strategies.

## Data Availability

Not applicable.

## Abbreviations

COPD: Chronic obstructive pulmonary disease
INESSS: Institut national d’excellence en santé et services sociaux (Quebec’s excellence institute in healthcare and social services)
ITS: Interrupted time series
KT: Knowledge translation
QI: Quality improvement
QIC: Quality improvement collaborative
QUAL: Qualitative
QUAN: Quantitative
